# The Polymorphisms in *GSTO* Genes (*GSTO1* rs4925, *GSTO2* rs156697, and *GSTO2* rs2297235) Affect the Risk for Testicular Germ Cell Tumor Development: A Pilot Study

**DOI:** 10.3390/life13061269

**Published:** 2023-05-27

**Authors:** Milos Petrovic, Tatjana Simic, Tatjana Djukic, Tanja Radic, Ana Savic-Radojevic, Milica Zekovic, Otas Durutovic, Aleksandar Janicic, Bogomir Milojevic, Boris Kajmakovic, Marko Zivkovic, Nebojsa Bojanic, Uros Bumbasirevic, Vesna Coric

**Affiliations:** 1 Clinic of Urology, University Clinical Center of Serbia, 11000 Belgrade, Serbia; milospet93@gmail.com (M.P.); odurutovic@gmail.com (O.D.); aleksandarmjanicic@gmail.com (A.J.); em2bogomir@yahoo.com (B.M.); mockay@gmail.com (B.K.); bojanicnebojsa@gmail.com (N.B.); 2 Institute of Medical and Clinical Biochemistry, Faculty of Medicine, University of Belgrade, 11000 Belgrade, Serbia; tatjana.simic@med.bg.ac.rs (T.S.); tatjana.djukic@med.bg.ac.rs (T.D.); ana.savic-radojevic@med.bg.ac.rs (A.S.-R.); 3 Faculty of Medicine, University of Belgrade, 11000 Belgrade, Serbia; 4 Department of Medical Sciences, Serbian Academy of Sciences and Arts, 11000 Belgrade, Serbia; 5 Institute of Mental Health, 11000 Belgrade, Serbia; tanjajevtic@gmail.com; 6 Centre of Research Excellence in Nutrition and Metabolism, Institute for Medical Research, National Institute of Republic of Serbia, University of Belgrade, 11000 Belgrade, Serbia; zekovicmilica@gmail.com

**Keywords:** testicular GCT, glutathione transferase, *GSTO*, gene polymorphism, oxidative stress

## Abstract

Members of the omega class of glutathione transferases (GSTs), GSTO1, and GSTO2, catalyze a range of reduction reactions as a part of the antioxidant defense system. Polymorphisms of genes encoding antioxidant proteins and the resultant altered redox profile have already been associated with the increased risk for testicular germ cell cancer (GCT) development. The aim of this pilot study was to assess the individual, combined, haplotype, and cumulative effect of *GSTO1*rs4925, *GSTO2*rs156697, and *GSTO2*rs2297235 polymorphisms with the risk for testicular GCT development, in 88 patients and 96 matched controls, through logistic regression models. We found that carriers of the GSTO1*C/A*C/C genotype exhibited an increased risk for testicular GCT development. Significant association with increased risk of testicular GCT was observed in carriers of *GSTO2*rs2297235*A/G*G/G genotype, and in carriers of combined *GSTO2*rs156697*A/G*G/G and *GSTO2*rs2297235*A/G*G/G genotypes. Haplotype H7 (*GSTO1*rs4925*C/*GSTO2*rs2297235*G/*GSTO2*rs156697*G) exhibited higher risk of testicular GCT, however, without significant association (*p* > 0.05). Finally, 51% of testicular GCT patients were the carriers of all three risk-associated genotypes, with 2.5-fold increased cumulative risk. In conclusion, the results of this pilot study suggest that *GSTO* polymorphisms might affect the protective antioxidant activity of GSTO isoenzymes, therefore predisposing susceptible individuals toward higher risk for testicular GCT development.

## 1. Introduction

Germ cell tumors (GCTs) are a diverse cluster of malignancies most frequently arising in the gonads (both testes and ovaries), and rarely at extragonadal sites on the body’s central axis supposedly following the migration course of the primordial germ cells [1]. It is estimated that 95% of testicular cancers have a germ cell origin. While being regarded as relatively rare oncopathologies in the general population, testicular malignancies are the most prevalent type of solid tumors among adolescents and young male adults, and the dominant cause of cancer-related mortality and morbidity in this population cohort [2]. Although demonstrating a pronounced geographical and ethnic variation, the incidence of testicular GCTs has been continuously increasing on a global level since the middle of the 20th century [3].

Displaying a wide array of histopathological profiles, diverse oncogenic trajectories, and clinical behaviors with pertinent diagnostic and treatment implications, testicular GCTs are broadly assorted into two major categories—seminoma and nonseminoma— with multiple additional categories of both embryonic and extraembryonic lineage, distinguishable by morphopathologic and immunohistochemical characteristics [4]. Based on current epidemiological evidence, an intricate interplay between the (epi)genetic constitution and environmental factors, labeled as the “genvironmental hypothesis”, has been implicated in testicular GTC predisposition, and is considered to contribute to these rising trends. The aforementioned parameters, affecting a plethora of signaling pathways and thereby developmental processes, may cause aberrant regulation of germ cell proliferation and maturation, resulting in the malignant transformation with diverse clinical and phenotypical characteristics [5]. Previous multidisciplinary studies investigating the origin of these tumors denoted germ cell neoplasia in situ as a common precursor lesion for invasive tumors in adolescents and young adults, contrary to prepubertal cases [4]. The alteration of germ cell differentiation is related to testicular dysgenesis syndrome, representing disturbed fetal development of male gonads with variable severity and manifestation. Numerous elements of this complex syndrome encompassing cryptorchidism, i.e., unilateral or bilateral undescended testes, abnormal spermatogenesis, altered fertility, positive family history of testicular cancer among first-degree relatives, hypospadia, and current or previous diagnosis of a contralateral tumor or germ cell neoplasia in situ, have been correlated with the increased risk of GCTC [6,7]. Additionally postulated risk factors comprise a spectrum of individual determinants (genetic aberrations, personal health and lifestyle-associated features, and professional and other hazardous exposures), together with certain maternal and environmental determinants [3]. 

Although there is an undisputable knowledge evolution in this field of urooncology, the relatively young age of patients, increasing worldwide prevalence, and the overall burden of the disease impel further experimental and epidemiological research, enabling an even better understanding of relevant oncogenetic and biological drivers of testicular carcinogenesis. A complex cellular system featuring sophisticated regulation of diverse biochemical and genetic mechanisms is implicated in maintaining an equilibrium between the relative abundance of reactive species and antioxidants [8]. Glutathione S-transferases (GSTs) represent a large family of multifunctional proteins [9,10], constituting the first line of antioxidant defense along with glutathione peroxidase (GPX) and catalase [11]. Moreover, GSTs have proved themselves as valuable biomarkers of risk and prognosis for diseases hallmarked with disturbed redox homeostasis, such as cancers [12,13,14].

The family of cytosolic GSTs comprises seven classes, including the omega class represented by two members, GSTO1-1 and GSTO2-2 [9]. These isoenzymes are remarkably distinctive from other members of the GST cytosolic family, especially in terms of active site structure (containing cysteine rather than tyrosine or serine), which renders them less performative in catalyzing conjugation reactions of certain compounds to glutathione, GSH [15,16,17]. Due to this particular feature, GSTO isoenzymes are involved in a range of reduction reactions with substrates that are rather uncharacteristic for other GSTs [16,17]. The underlying mechanisms of such novel reduction reactions comprise the ability of GSTO1 to recuperate thiol groups of certain proteins upon their oxidative damage. In this tightly regulated thiol transferase reaction, GSTO1 uses free GSH for deglutathionylation, rather than for conjugation with electrophilic compounds [18,19]. On the other hand, GSTO2 exhibits very high GSH-dependent dehydroascorbate reductase activity, which is considered important in preserving ascorbate levels [17,18,20]. What is more, GSTO2 has remarkably high expression in the testis [17,21]. 

The data provided lend support to the members of the omega GST class being involved in maintaining the cellular redox homeostasis. Their polymorphic expression may diminish their catalytic properties and take a toll on their antioxidant protection capacities. In the case of GSTO isoenzymes, three particular polymorphisms (*GSTO1*rs4925, *GSTO2*rs156697, and *GSTO2*rs2297235; Supplementary Figure S1) have been investigated in various clinical scenarios, especially as new perspective candidates for risk biomarkers in the field of urologic oncology [22,23,24,25,26]. Indeed, the widely assessed polymorphisms are associated with their reduced reduction activity, as *GSTO1* single nucleotide polymorphism (GSTO1*C419A (rs4925)) affects the deglutathionylase and thioltransferase activity. Namely, the GSTO1*A allele has lower deglutathionylase activity and higher activity in the forward glutathionylation reaction, compared to the wild-type GSTO1*C allele [10,17,27,28]. On the other hand, GSTO2*A424G (rs156697) affects its protein levels [10,28,29]. What is more, strong linkage disequilibrium has been identified for these polymorphisms, including the GSTO2*A183G, rs2297235, placed within the 5′ untranslated (5′ UTR) gene region [30]. 

A wide range of factors has been suspected of being associated with the etiopathology of testicular germ cell tumor (GCT), including individual genetic alterations, intensifying the predisposition to cancer development [7]. Polymorphisms occurring within genes encoding the antioxidant proteins have been assessed in our previous study, investigating the inter-individual susceptibility toward testicular GCT development in patients with presumably altered redox profile [31]. As reactive species may set an ambient for cancer onset and evolution, the redox status of still very young male individuals could be additionally characterized by perturbations due to reduced antioxidant activities of GSTO isoenzymes. Several polymorphisms in the GST omega class have been acknowledged as cancer-risk biomarkers [22,23,24,25], but, to the best of our knowledge, this is the first study investigating the association of *GSTO1* polymorphic expression in relation to testicular tumorigenesis [31,32,33,34,35]. Therefore, the aim of this pilot study was to further define a unique redox profile by determining the individual, combined, haplotype, and cumulative effect of *GSTO1*rs4925, *GSTO2*rs156697, and *GSTO2*rs2297235 genetic variants on the risk for testicular germ cell tumor development.

## 2. Materials and Methods

### 2.1. Study Population

Our case-control study consecutively recruited 88 patients (average age 33.5 ± 8.7 years) with newly diagnosed testicular GCT, treated at the Clinic of Urology, University Clinical Centre of Serbia, Belgrade, between 2020 and 2021 [31,36]. Following preoperative diagnostics, which consisted of physical examination, ultrasonographic and radiographic assessment, and evaluation of serum tumor markers (alpha-fetoprotein (AFP), beta-human chorionic gonadotropin (BHCG), and lactate dehydrogenase (LDH)), histopathological diagnosis of testicular GCT was established by an experienced uropathologist, employing the WHO classification [37]. Patients younger than 18 years and those with a previous medical history of cancer or ongoing oncological treatment for other malignancies were excluded from the study. All study participants provided their informed consent for participating in this pilot study. Data regarding their demographics, personal histories, and diagnostic and treatment procedures were acquired via standardized questionnaire and medical records. The control group included 96 (average age 35.8 ± 9.7 years) age-matched subjects, without previous history of cancer, whose DNA samples are stored in the DNA biobank at the Institute of Medical and Clinical Biochemistry, Faculty of Medicine, University of Belgrade. The potential confounding effect of geographic location and ethnic origin was decreased by recruiting the control participants within the same population as the cases. 

### 2.2. Ethical Approval 

The pilot research was carried out in accordance with the standards of the Helsinki Declaration as well as with the Ethics Board of University Clinical Centre of Serbia, Serbia.

### 2.3. GSTO Genotyping

Genomic DNA was isolated from preoperatively obtained whole blood EDTA samples, employing a commercial kit (The PureLink™ Gel Extraction Kit # K210025, Invitrogen, Waltham, MA). GSTO1*C419A (rs4925) (ID: C_11309430_30), GSTO2*A424G (rs156697) (ID:C_3223136_1), and GSTO2*A183G (rs2297235) (ID: C_3223142_1) genotypes were determined by quantitative polymerase chain reaction (qPCR), using TaqMan SNP Genotyping assays (Life Technologies, Applied Biosystems, Carlsbad, CA, USA) along with Maxima™ Hot Start Master mix and distilled water (Thermo Fisher Scientific, Waltham, MA, USA), according to the manufacturer’s instructions. The amplification reaction consisted of 40 repeated cycles of appropriate thermal protocols. The reaction was monitored, and obtained results analyzed by Mastercycler ep realplex software (Eppendorf, Hamburg, Germany). 

### 2.4. Statistical and Haplotype Analysis

Statistical analysis was performed using the Statistical Package for the Social Sciences (SPSS, version 17, SPSS Inc., Chicago, IL, USA). The χ^2^ test was used to evaluate the differences between categorical variables, and to determine if the genotype distribution deviated from Hardy–Weinberg equilibrium. Logistic regression analysis was used to explore the association between the GSTO genetic variants and the risk for testicular GCT development by determining odds ratios (OR) and 95% confidence intervals (CI). The initial analysis was performed in order to establish risk-associated genotypes throughout several models. Based on the availability of a sufficient number of the alleles and genotypes, the effect of the combined, haplotype, and cumulative analysis of *GSTO1*rs4925, *GSTO2*rs156697, and *GSTO2*rs2297235 genetic variants was estimated through proposed models, when possible. Overall, two risk models were calculated: model 1 without any adjustments (crude OR1) and model 2 adjusted to the other two remaining genotypes (OR2). Haploview (version 4.1, Broad Institute, MIT, Harvard, MA, USA) was used to assess the extent of linkage disequilibrium (LD) between pairs of SNPs [38]. The effect of different GSTO haplotypes on the risk for testicular GCT development was confirmed using SNPStats [39]. A *p* value ≤ 0.05 was considered to be statistically significant.

## 3. Results

The demographic and clinico-pathological characteristics of 88 patients with testicular GCT are shown in Table 1.

The genotype distribution of GSTO1*C419A (rs4925), GSTO2*A424G (rs156697), and GSTO2*A183G (rs2297235) in control subjects was in Hardy–Weinberg equilibrium (*p* > 0.05). The distributions of *GSTO* gene polymorphisms and the risk for the development of testicular GCT are presented in Table 2. The individual effect of GSTO1*C419A (rs4925), GSTO2*A424G (rs156697), and GSTO2*A183G (rs2297235) polymorphisms on the risk for testicular GCT development was assessed in order to establish risk-associated genotypes. For this purpose, the GSTO1*A allele was regarded as the reference group throughout the analysis, as opposed to the GSTO1*C allele, which is otherwise known as the wild-type allele.

Although the carriers of GSTO1*C/A*C/C genotype exhibited an increased risk for testicular GCT development (OR1 = 2.14, Table 2) in comparison with the carriers of the referent genotype GSTO1*A/A, statistically significant association with increased risk of testicular GCT was observed in Model 2 (OR2 = 3.20; 95% CI: 1.1–9.35; *p* = 0.033; Table 2). When the individual modifying effect of GSTO2*A424G (rs156697) on testicular GCT risk development was assessed, patients who carried the GSTO2*A/G*G/G genotype were running the higher risk; however, this association did not reach statistical significance in both assessed models (*p* > 0.05, Table 2). Regarding the GSTO2*A183G (rs2297235) gene polymorphism, an increased risk was observed in carriers of the GSTO2*A/G*G/G genotype in Model 1 of logistic regression analysis (OR1 = 1.92; 95% CI: 1.06–3.47; *p* = 0.031; Table 2) when compared to the individuals with the referent genotype (GSTO2*A/A). Due to the biological origin and the clinically distinctive characteristics of the two large categories of testicular GCT, the individual effect of GSTO1*C419A (rs4925), GSTO2*A424G (rs156697), and GSTO2*A183G (rs2297235) polymorphisms on the risk for seminoma development was estimated as well (Supplementary Table S1). As the distribution of the GSTO2*A424G (rs156697) genotypes differed from the whole group of testicular GCT patients, the association of the *GSTO2*rs156697*A/G*G/G genotype did reach statistical significance in Model 1 (OR1 = 2.24; 95% CI: 1.09–4.60; *p* = 0.028; Supplementary Table S1). 

Additional efforts were invested in addressing the potential combined effect. Since there were no recruited cases or controls carrying all three referent genotypes (*GSTO1*rs4925*A/A, *GSTO2*rs2297235*A/A, and *GSTO2*rs156697*A/A), we examined the combined effect of only *GSTO2*rs156697 and *GSTO2*rs2297235 gene polymorphisms on the risk for testicular cancer development. Results are shown in Table 3. Individuals with combined *GSTO2*rs156697*A/G*G/G and *GSTO2*rs2297235*A/G*G/G genotypes had 2.5-fold higher risk for testicular GCT development in comparison with referent genotype combination (OR2 = 2.49; 95% CI: 1.25–4.96; *p* = 0.010). 

**Figure 1:**
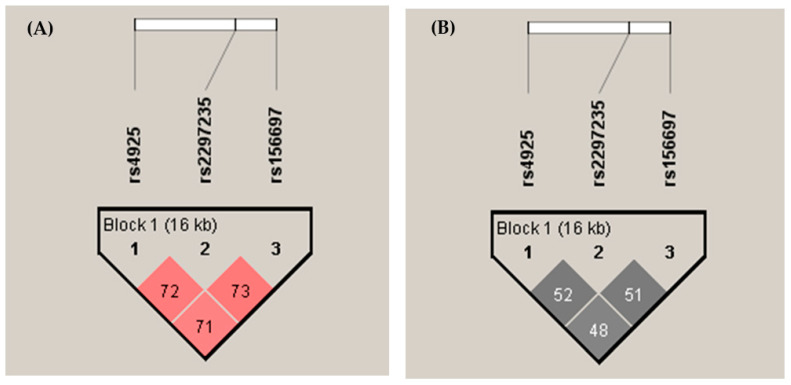
LD plots: D’/LOD standard (**A**) and r^2^—correlation coefficient between the two loci (**B**) for GSTO1*C419A (rs4925), GSTO2*A424G (rs156697), and GSTO2*A183G (rs2297235) polymorphisms; Exported from Haploview [38].

The effect of different *GSTO* haplotypes was estimated through the non-random association of *GSTO* alleles and expressed as the normalized coefficient of LD (D’, Table 4). Since D’ values can range from 0 to 1.0, a value of 1.0 indicates that two polymorphisms are maximally associated, whereas 0 indicates they are randomly associated [40]. We found a D’ of 0.71 between *GSTO1*rs4925 and *GSTO2*rs156697, 0.725 for *GSTO1*rs4925 and *GSTO2*rs2297235, and 0.73 between *GSTO2*rs156697 and *GSTO2*rs2297235 (Figure 1A), confirming a high LD between these SNPs (*p* < 0.001). In addition, the correlation coefficient (r^2^) between the two loci was around 0.5 for all three assessed polymorphisms (Figure 1B). Moreover, the statistical analysis indicated that the haplotype H7 (*C*G*G) exhibited three-fold higher risk of testicular GCT; however, there was no significant association (OR = 3.37; 95% CI: 0.60–18.78; *p* = 0.174; Table 4). No significant association was obtained when adjusted haplotype analysis was performed. 

Since it was not manageable to assess the combined effect of all the analyzed genotypes, we calculated the cumulative effect of all three polymorphisms by adding the previously established *GSTO* risk-associated genotypes (*GSTO1*rs4925*C/A*C/C, *GSTO2* rs2297235*A/G*G/G, *GSTO2*rs156697*A/G*G/G; Table 2). Half of the recruited patients (51%) were the carriers of all three risk-associated genotypes and exhibited 2.5-fold increased risk for testicular GCT development (OR1 = 2.53; 95% CI: 1.27–5.04; *p* = 0.008; Table 5). 

## 4. Discussion

As proposed earlier, single nucleotide polymorphisms (SNPs) occurring within genes encoding for antioxidant proteins might lead to disturbed antioxidant defense, promoting oxidative stress and contributing to the risk for tumor development, including testicular cancer [31]. Therefore, in this pilot study, we made an attempt to further define a unique redox profile by determining the individual, combined, haplotype, and cumulative effect of *GSTO1*rs4925, *GSTO2*rs156697, and *GSTO2*rs2297235 genetic variants on the risk for testicular GCT development, by conducting two logistic regression risk models. Additional calculations were performed regarding the assessment of the risk for seminoma development. We found that the carriers of GSTO1*C/A*C/C genotype exhibited an increased risk for testicular GCT development. Similarly, the significant association with increased risk of testicular GCT was observed in carriers of *GSTO2*rs2297235*A/G*G/G genotype, and in carriers of combined *GSTO2*rs156697*A/G*G/G and *GSTO2*rs2297235*A/G*G/G genotypes. Haplotype H7 (*GSTO1*rs4925*C/*GSTO2*rs2297235*G/*GSTO2*rs156697*G) exhibited higher risk of testicular GCT, however, without significant association. Finally, 51% of testicular GCT patients were the carriers of all three risk-associated genotypes, with 2.5-fold increased cumulative risk. Interestingly, the carriers of *GSTO2*rs156697*A/G*G/G genotype exhibited increased risk for seminoma development. 

GSTs hold an unprecedented role within the second phase of cellular detoxification reactions [9,41,42]; however, they have been recognized as regulators of protein activity, cell proliferative capacity, and survival signaling pathways as well [10,43]. Omega class members (GSTO) exert both catalytic and non-catalytic roles. Firstly, given the specific structural and functional properties, GSTO1 may provide a valuable contribution to defense against irreversible oxidative damage of redox-sensitive protein thiol groups by involvement in the glutathionylation cycle and glutaredoxine-like activity [18,19]. In addition, GSTO2 catalyze dehydroascorbate reductase (DHAR) reactions further contribute to the maintenance of cellular redox homeostasis [18]. Due to the aforementioned structural features, GSTO1 are specific toward larger and less hydrophobic substrates, unlike other GSTs [16,20]. Some of the most recognized comprise glutathione-dependent reduction reactions of pentavalent methylated arsenic species, monomethylarsenate^V^ and dimethylarsenate^V^ [17,44,45]. Arsenic is known for its high toxicity and carcinogenicity, raising the risk for bladder, lung, kidney, and liver cancer development [46]. Unfortunately, the environmental and occupational exposure to aforementioned arsenic compounds in the association with glutathione S-transferase O1 and O2 polymorphisms could not be assessed in this pilot study. Although the epidemiological data regarding the exposure to endocrine-disrupting chemicals (such as organochlorine pesticides) and the risk of male reproductive disorders are limited, some of these compounds have been previously linked to breast cancer development in carriers of other *GST variant* genotypes [47] but not with *GSTO* genotypes. Moreover, there are no available data on human GST omega expression and activity in response to organochlorine exposure, even though the retinoid X receptor α (RXRα) and Pregnane X receptor (PXR) pathways [48] are considered to be master xenobiotic receptors implicated in coordinately regulating the genes encoding drug-metabolizing enzymes, such as GSTs. Finally, GSTO1 has several non-catalytic roles, comprising the modulation of posttranslational processing of pro-IL1β to its active form and modulation of ryanodine receptors [49,50]. In addition, anti-apoptotic and pro-survival role of GSTO1 was acknowledged as an important part of cellular chemoresistance strategy in some cancer cell lines [51,52].

Noteworthy gene heterogeneity was observed within GST omega class members, comprising more than 30 polymorphisms within the *GSTO1* gene and more than 60 polymorphisms in the *GSTO2* gene [53]. *GSTO1*rs4925, *GSTO2*rs156697, and *GSTO2*rs2297235 polymorphisms have already been credited as risk biomarkers, since they were associated with susceptibility to various cancers [25,30,54,55,56,57,58,59]. Indeed, the results of our pilot study showed that the carriers of *GSTO1*rs4925*C/A*C/C genotype were more predisposed to testicular GCT development in comparison with the carriers of the referent *GSTO1*rs4925*A/A genotype. Still, the carriers of this particular genotype exhibited increased risk for seminoma development, however, without significant calculation. GSTO1 regulates the function of certain proteins by catalyzing both the glutathionylation and deglutathionylation, through the so-called glutathionylation cycle, described by Board and Menon [10]. In addition, it has been shown that the variant GSTO1*A allele exhibits lower deglutathionylase activity and higher activity in the forward glutathionylation reaction, as opposed to the GSTO1*C wild-type allele [10,28]. Therefore, this particular SNP might affect GSTO1 specificity toward particular proteins or particular glutathionylated cysteine residues, affecting the posttranslational regulation of proteins, especially those involved in tumor growth [59], shedding some light on the possible link between *GSTO1*rs4925 polymorphism and different cancers. To the best of our knowledge, this is the first study investigating the association of *GSTO1* polymorphic expression with testicular GCT development. Thus far, none of the studies have assessed the effect of *GSTO1* genetic variations on their antioxidant activity within testicular tissue. 

Vitamin C represents an essential hydrophilic component of the antioxidant defense system, scavenging free radicals and specific reactive oxygen species. As a principal antioxidant in the testis, it seems to be indispensable in protecting the testicular cells’ integrity and components from oxidative damage, at all stages of their development and maturation [60,61]. Indeed, the abnormalities in germ cell development and migration, taking place during testicular GCT evolution, are still of unclear etiology. The majority of the contributing risk factors, occurring in pre/peri/postnatal age, are a part of the aforementioned “genvironmental hypothesis” puzzle [7] and can be associated with disturbed redox homeostasis [62]. Dehydroascorbate and ascorbyl radicals are readily recycled into ascrobate through mechanisms comprising the direct reduction by glutathione, and enzymatic reduction by various thiol transferases and NADPH-dependent reductases [21,63]. Human omega-class GSTs play a pivotal role in this reductive biochemistry, with GSTO2 having the highest dehydroascorbate reductase activity within mammalian systems [17,44,64]. What is more, GSTO2 has remarkably high expression in the testis [17,21]. The aforementioned recycling mechanisms can be altered in a chronically disturbed redox homeostasis and associated with impaired ascorbate concentrations. In this pilot study, we assessed the SNP polymorphism of GSTO2*A424G (rs156697), presumed to primarily affect its antioxidant dehydroascorbate reductase activity and thereby ascorbate preservation [20,29]. Indeed, the carriers of the GSTO2*G allele (rs156697) were in particular at higher risk of developing seminoma compared to the carriers of *GSTO*2*AA genotype. As far as *GSTO2*rs2297235 polymorphism is concerned, we found that the carriers of the GSTO2*A/G*G/G genotype exhibited increased risk for testicular GCT and seminoma development, as opposed to those with the GSTO2*AA genotype. Lowered dehydroascorbate reductase activity in individuals with both variant *GSTO2* alleles might result in deficient recycling mechanisms of vitamin C and accumulation of dehydroascorbate [17,44,65], contributing to the disruption of redox homeostasis. What is more, when *GSTO2* genotypes (rs156697 and rs2297235) were analyzed in combination, the obtained results confirmed that the combined GSTO2*A/G*G/G and GSTO2*A/G*G/G genotype was associated with a significantly increased risk of testicular GCT. 

The results of this pilot study demonstrated significant linkage disequilibrium of GSTO polymorphisms. Therefore, we additionally evaluated the effect that *GSTO* haplotypes have on testicular GCT risk. Haplotype H7 (*GSTO1*rs4925*C/*GSTO2*rs2297235*G/*GSTO2*rs156697*G) exhibited three-times higher risk of testicular GCT, however, without significant association. The results of this pilot study may serve in identifying the haplotype that would predispose such carriers toward urological cancers. Interestingly, the carriers of the very same haplotype exhibited increased risk of bladder cancer [30] and clear renal cell carcinoma development [22], whereas the findings by Djukic et al. confirmed the association of the *GSTO1*rs4925*C/ *GSTO2*rs156697*G haplotype with higher risk of bladder cancer [25]. 

Due to the overlapping GSTO isoenzyme activities [16], it was important to analyze the cumulative effect of all *GSTO* genotypes, as the impaired activity of the single given GSTO may be compensated by the other one. Surprisingly, we observed the risk-associated combination featuring *GSTO1*rs4925*C/A*C/C + *GSTO2*rs2297235*A/G*G/G + *GSTO2*rs156697*A/G*G/G genotypes in 51% of all the participating testicular GCT patients. This cumulative effect together with combined and haplotype effect of *GSTO* polymorphisms is most certainly associated with altered deglutathionylase and dehydroascorbate reductase activity, additionally disturbing the redox homeostasis and influencing the susceptibility for testicular GCT development [31].

The present pilot study has certain limitations that should be acknowledged. Firstly, due to inherent methodological drawbacks of the case-control design, potential selection bias might undermine the robustness and validity of the observed findings. Nevertheless, this research approach is commonly applied in candidate–gene association studies, and is deemed particularly practical for the investigation of risk-associated genetic variants for uncommon pathologies such as testicular GCT. Furthermore, the absence of cases or controls carrying all three non-risk associated genotypes (*GSTO1*rs4925*A/A + *GSTO2* rs2297235*A/A+ *GSTO2*rs156697*A/A) may be attributed to relatively small sample size. Consequently, the combined effect and the total cumulative effect of all three genotypes could not be assessed. Given that transcriptome analysis was beyond the research scope of the present pilot study, and due to the general paucity of such data, additional systematic transcriptomic investigations are needed for an in-depth portrayal of *GSTO* gene signatures, their aberrant expression affecting testicular carcinogenesis, and the potential detection of molecular targets relevant to diagnostic or intervention strategies. Finally, the interpretation of the presented conclusions should be exercised with reasonable caution and recognition of ethnic and racial-related discrepancies and gene-environmental interactions that may affect the replicability of the reported results in other populations. Therefore, multicentric, larger-scale research endeavors are warranted to further explore the underlying biological mechanisms, clinical relevance, and magnitude of the GSTO-associated testicular cancer susceptibility risk. 

## 5. Conclusions

Certain polymorphisms in *GSTO* genes (*GSTO1*rs4925, *GSTO2*rs156697, and *GSTO2*rs2297235) modulate the risk for testicular GCT development. To the best of our knowledge, this is the first study to analyze the individual, combined, haplotype, and cumulative effect of these well-known *GSTO* SNPs. Having in mind the antioxidant activities of GSTO1 and GSTO2, the results obtained on the effect of *GSTO1*rs4925, *GSTO2* rs156697, and *GSTO2*rs2297235 genetic variants on the risk for testicular GCT development imply the importance of these SNPs in terms of inter-individual susceptibility to oxidative stress, further defining a unique redox profile in such young population of patients. Although the proposed causal flow of investigated polymorphisms on the testicular GCT should be investigated in terms of validating the proposed sequence of events, at this stage we presented the model that will be further explored in subsequent studies due to the current unavailability of data reflecting the antioxidant activity of GSTO isoenzymes.

## Figures and Tables

**Table 1:** The characteristics of testicular GCT patients.

Parameters	Testicular GCT Patients
Age (years)	33.5 ± 8.7 ^1^ 33.5 (19–54) ^2^
Risk factors for testicular GCT development, n (%)	
Cryptorchismus	8 (9)
Infertility	4 (5)
Testicular atrophy	7 (8)
Testicular dysgenesis syndrome	1 (1)
Positive family history	4 (5)
Tumor type	
Seminoma	52 (59)
Non-seminoma	36 (41)
Clinical stage	
I	61 (69)
II	18 (21)
III	9 (10)
Pathological stage	
pT1	40 (47)
pT2	39 (46)
pT3	5 (6)
pT4	1 (1)

^1^ mean ± standard deviation; ^2^ median (min–max).

**Table 2:** The individual effect of GSTO1*C419A (rs4925), GSTO2*A424G (rs156697), and GSTO2*A183G (rs2297235) polymorphisms on the risk for testicular GCT development.

Genotype	Patients n (%)	Controls n (%)	Crude OR1 ^1^ (95% CI)	*p*	OR2 ^2^ (95% CI)	*p*
*GSTO1*rs4925 ^3^						
*A/A	6 (7)	13 (14)	1.00	-	1.00 (reference group)	-
*C/A*C/C	81 (93)	82 (86)	2.14 (0.77–5.90)	0.142	3.20 (1.1–9.35)	0.033
*GSTO2*rs156697 ^4^						
*A/A	33 (39)	50 (52)	1.00	-	1.00 (reference group)	-
*A/G*G/G	51 (61)	46 (48)	1.68 (0.93–3.04)	0.087	1.34 (0.59–3.27)	0.455
*GSTO2*rs2297235 ^5^						
*A/A	31 (35)	49 (51)	1.00	-	1.00 (reference group)	-
*A/G*G/G	57 (65)	47 (49)	1.92 (1.06–3.47)	0.031	1.80 (0.75–4.27)	0.186

In order to establish risk-associated genotypes, the GSTO1*A allele was regarded as reference group throughout the analysis, as opposed to the wild-type allele, GSTO1*C allele. ^1^ OR1, crude odds ratio; ^2^ OR2 adjusted to other two remaining genotypes; ^3^ For *GSTO1*rs4925 genotyping was successful in 99% of patients and 100% of controls; ^4^ *GSTO2*rs156697 genotyping was successful in 95% of patients and 100% of controls; ^5^ *GSTO2*rs2297235 genotyping was successful in 100% of patients and 100% of controls; CI—confidence interval; 1.00—reference group.

**Table 3:** The combined effect of GSTO2*A424G (rs156697) and GSTO2*A183G (rs2297235) polymorphisms on the risk for testicular GCT development.

*GSTO2*rs156697/ *GSTO2*rs2297235	Patients n (%)	Controls n (%)	Crude OR1 ^1^ (95% CI)	*p*	OR2 ^2^ (95% CI)	*p*
*A/A + *A/A	29 (34)	40 (42)	1.00	-	1.00	
*A/A + *A/G*G/G	4 (5)	10 (10)	0.55 (0.16–1.93)	0.354	0.59 (0.17–2.08)	0.413
*A/G*G/G + *A/A	2 (3)	9 (9)	0.37 (0.06–1.52)	0.149	0.31 (0.06–1.52)	0.149
*A/G*G/G + *A/G*G/G	49 (58)	37 (39)	1.83 (0.96–3.47)	0.065	2.49 (1.25–4.96)	0.010

^1^ OR1, crude odds ratio; ^2^ OR2, adjusted to other two remaining genotypes; CI—confidence interval; 1.00—reference group.

**Table 4:** The effect of GSTO1*C419A (rs4925), GSTO2*A183G (rs2297235), and GSTO2*A424G (rs156697) haplotypes on the risk for testicular GCT development.

Haplotype	*GSTO1* rs4925	*GSTO2* rs2297235	*GSTO2* rs156697	Frequency	Crude OR (95% CI)	*p*
H1	*C	*A	*A	0.563	1.00	-
H2	*A	*G	*G	0.245	1.30 (0.79–2.14)	0.312
H3	*A	*A	*A	0.040	0.54 (0.15–1.94)	0.355
H4	*C	*G	*A	0.034	0.73 (0.22–2.42)	0.619
H5	*C	*A	*G	0.037	1.75 (0.52–5.87)	0.374
H6	*A	*A	*G	0.022	0.27 (0.04–1.67)	0.166
H7	*C	*G	*G	0.027	3.37 (0.60–18.78)	0.174
H8	*A	*G	*A	0.032	0.41 (0.07–2.31)	0.315

SNPStats global haplotype association *p*-value: 0.140; Crude OR—crude odds ratio; CI—confidence interval; 1.00—reference group.

**Table 5:** The cumulative effect of GSTO1*C419A (rs4925), GSTO2*A424G (rs156697), and GSTO2*A183G (rs2297235) polymorphisms on the risk for testicular GCT development.

*GSTO1*rs4925/*GSTO2*rs156697/*GSTO2*rs2297235	Patients n (%)	Controls n (%)	Crude OR1 ^1^ (95% CI)	*p*
1	29 (34)	41 (43)	1.00 (reference group)	
2	12 (14)	30 (32)	0.57 (0.24–1.28)	0.174
3	43 (51)	24 (25)	2.53 (1.27–5.04)	0.008

^1^ crude odds ratio; CI—confidence interval; 1.00—reference group. 1, 2, 3: number of the risk-associated genotypes present: either one of each risk-associated, or two of each risk-associated, or all three risk-associated *GSTO* genotypes (*GSTO1*rs4925*C/A*C/C+ *GSTO2* rs2297235*A/G*G/G + *GSTO2*rs156697*A/G*G/G). There were no recruited cases or controls carrying all three referent genotypes (*GSTO1*rs4925*A/A + *GSTO2*rs2297235*A/A+ *GSTO2*rs156697*A/A), which would have been marked by zero (0).

## Data Availability

The data supporting reported results can be found upon request in the form of datasets available at Clinic of Urology, University Clinical Centre of Serbia and LymeSurvey repository, https://upitnik.med.bg.ac.rs/, accessed on 4 February 2023, Institute of Medical and Clinical Biochemistry, Faculty of Medicine, University of Belgrade.

## Supplementary Materials

The following supporting information can be downloaded at: https://www.mdpi.com/article/10.3390/life13061269/s1, Figure S1. Observation of the investigated polymorphisms in *GSTO1* and *GSTO2* genes indicating the SNPs location and proposed effects; Table S1. The individual effect of GSTO1*C419A (rs4925), *GSTO2**A424G (rs156697), and *GSTO2**A183G (rs2297235) polymorphisms on the risk for seminoma development.
