# Supplementary material for: The Polymorphisms in GSTO Genes (GSTO1 rs4925, GSTO2 rs156697, and GSTO2 rs2297235) Affect the Risk for Testicular Germ Cell Tumor Development: A Pilot Study
Source: Life (Basel). 2023 May 27;13(6):1269. doi: 10.3390/life13061269 (PMC10301167; doi:10.3390/life13061269)
Supplement: Supplementary file 1 [file life-13-01269-s001.zip › life-2254896-supplementary.pdf]

Supplementary Materials

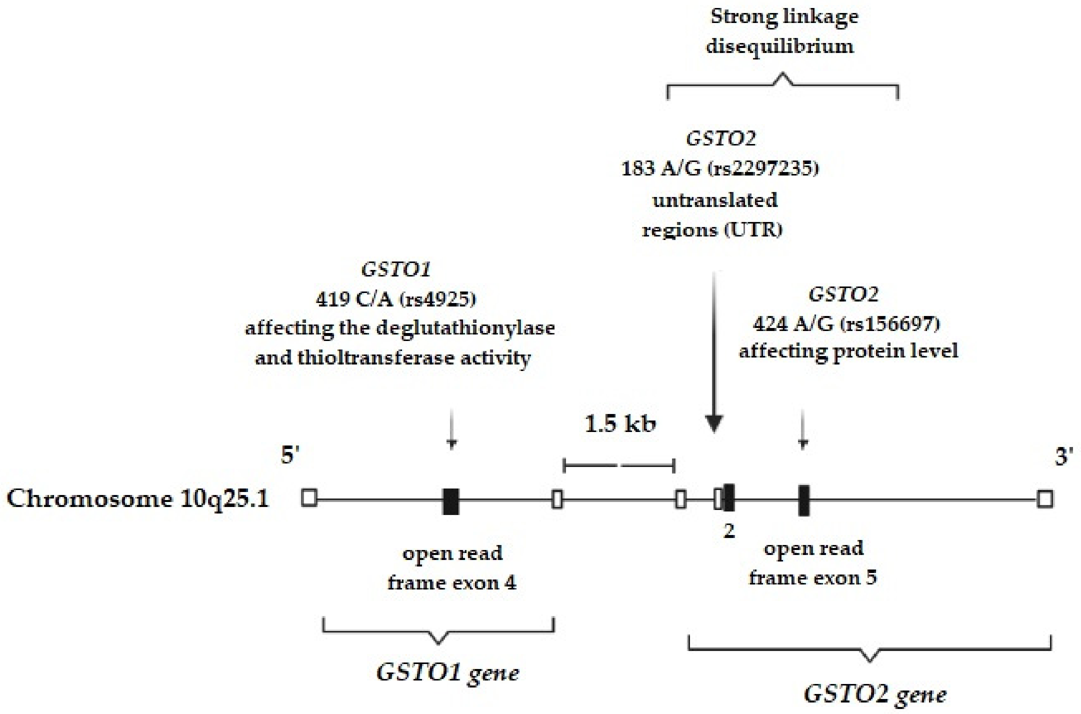

Figure S1. Observation of the investigated polymorphisms in *GSTO1* and *GSTO2* genes indicating the SNPs location and the proposed effects

Table S1. The individual effect of *GSTO1**C419A (rs4925), *GSTO2**A424G (rs156697), and *GSTO2**A183G (rs2297235) polymorphisms on the risk for seminoma development

Genotype	Patients with Seminoma n (%)	Controls n (%)	Crude OR1 [1] (95% CI)	*p*	OR2 [2] (95% CI)	*p*
*GSTO1*rs4925						
*A/A	4 (8)	13 (14)	1.00	-	1.00	-
*C/A*C/C	47 (92)	82 (86)	1.86 (0.57-6.04)	0.300	2.88 (0.83-9.93)	0.094
*GSTO2*rs156697						
*A/A	16 (33)	50 (52)	1.00	-	1.00	-
*A/G*G/G	33 (67)	46 (48)	2.24 (1.09-4.60)	0.028	2.34 (0.86-6.40)	0.095
*GSTO2*rs2297235						
*A/A	18 (35)	49 (51)	1.00	-	1.00	-
*A/G*G/G	34 (65)	47 (49)	1.97 (0.98-3.95)	0.057	1.25 (0.46-3.42)	0.657

[1] OR1 crude odds ratio; [2] OR2 adjusted to the other two remaining genotypes; CI- Confidence interval; 1.00- Reference group.
